# People have different expectations for their own versus others' use of AI‐mediated communication tools

**DOI:** 10.1111/bjop.12727

**Published:** 2024-09-04

**Authors:** Zoe A. Purcell, Mengchen Dong, Anne‐Marie Nussberger, Nils Köbis, Maurice Jakesch

**Affiliations:** ^1^ LaPsyDÉ Université Paris Cité, CNRS Paris France; ^2^ Center for Humans and Machines Max Planck Institute for Human Development Berlin Germany; ^3^ Cornell Tech Cornell University New York New York USA; ^4^ Department of Media Bauhaus University Weimar Germany

**Keywords:** AI‐mediated communication, artificial intelligence, computer‐mediated communication, human–AI interaction, technology risk assessment, trust

## Abstract

Artificial intelligence (AI) can enhance human communication, for example, by improving the quality of our writing, voice or appearance. However, AI mediated communication also has risks—it may increase deception, compromise authenticity or yield widespread mistrust. As a result, both policymakers and technology firms are developing approaches to prevent and reduce potentially unacceptable uses of AI communication technologies. However, we do not yet know what people believe is acceptable or what their expectations are regarding usage. Drawing on normative psychology theories, we examine people's judgements of the acceptability of open and secret AI use, as well as people's expectations of their own and others' use. In two studies with representative samples (Study 1: *N* = 477; Study 2: *N* = 765), we find that people are less accepting of secret than open AI use in communication, but only when directly compared. Our results also suggest that people believe others will use AI communication tools more than they would themselves and that people do not expect others' use to align with their expectations of what is acceptable. While much attention has been focused on transparency measures, our results suggest that self‐other differences are a central factor for understanding people's attitudes and expectations for AI‐mediated communication.

## BACKGROUND

Humans are social beings – our lives and identities are defined by our relationships. Our relationships depend on communication, assumptions of authenticity and interpersonal trust (Grueter & White, 2014; Hruschka, 2010). Artificial intelligence (AI) is radically transforming the way we communicate (Hancock et al., 2020; Sundar, 2020; Sundar & Lee, 2022). AI technologies that modify, augment or generate interpersonal communication have the potential to improve the efficacy of our communication (Hancock et al., 2020). However, they also have the potential to increase deception, threaten our perceptions of others' authenticity and promote mistrust (Jago, 2019; Jakesch, Hancock, & Naaman, 2023). Here, we review normative perspectives on the use of AI in communication and empirically describe people's perceptions of AI use in communication for themselves and for others and across settings where they are used openly or in secret.

AI is now involved in many communication experiences, such as with chatbots, voice assistants and video or image editors. The use of AI tools in interpersonal communication has been conceptualized as ‘AI‐mediated communication’ by Hancock et al. (2020) to highlight and theorize its effects on the communicative environments. People's expectations for AI‐mediated communication technologies—AI systems that modify messages to achieve *interpersonal communication goals*—are the focus of the current work. AI‐mediated communication technologies (hereafter, AI‐MCs) range from narrow‐AI text editors like Grammarly to multifunctional tools like ChatGPT and Capcut, are increasingly blurring the line between human and AI‐generated content (Jakesch, Hancock, & Naaman, 2023). In AI‐mediated communication, machines become active communication proxies or even delegates, paralleling observations in ethical studies indicating that AI systems become moral proxies that can blur moral intentions (Bonnefon et al., 2024; Purcell & Bonnefon, 2023b). As discussions about the ethics and implications of AI‐MCs evolve, understanding public perceptions and attitudes is crucial for guiding these conversations and regulations.

The rapid development of AI‐MCs has sparked lively debates on the risks and societal impact of emerging AI language technologies (Chesney & Citron, 2019; Hancock et al., 2020; Jakesch, Bhat, et al., 2023; Ruggeri, 2023). For example, initial studies have demonstrated their potential to increase deception and mistrust (Jakesch et al., 2019; Köbis et al., 2021). Both regulators and tech companies are trying to define, discourage and restrict the inappropriate use of AI‐generation tools (European Commission, 2024). For instance, the New York City school district was among the first to ban ChatGPT (Yang, 2023), Italy imposed a blanket ban (Browne, 2023) and Europol has raised grave concerns about its criminal potential (Chee, 2023), such as enabling new forms of phishing scams by hyperrealistic fraud impersonation. Although technical solutions, such as Large Language Model (LLM) detection algorithms, are being developed, new instances of effective deception are emerging. For example, this year an employee was scammed into wiring $25 million following a video conference in which all other attendees were deepfaked (Hsu, 2023). To facilitate more successful solutions to issues like these, it is critical that we understand how people interact with and are impacted by AI‐MCs. We contribute to this new field of investigation by evaluating public attitudes towards AI‐MC use.

In this article, we explore critical perceptions around the acceptability and expectations of AI‐MC use and pragmatic antecedents such as transparency (whether AI‐MCs are used openly or secretly) and the user perspective (considering our own or others' AI‐MC use). We focus on the self‐other distinction because people's perceptions of (1) what behaviours are acceptable or not (i.e., injunctive norms) and (2) what most other people typically do (i.e., descriptive norms) are both crucial factors that predict people's behaviours, especially those that are morally dubious (Bicchieri & Xiao, 2009). Moreover, people do not impose identical moral standards on themselves versus others (Valdesolo & DeSteno, 2007; Weiss et al., 2018), nor in public versus private settings (Vogt et al., 2016).

Social norms theory outlines that social beliefs create a shared understanding of (un)acceptable behaviours and have important implications on the proper functioning of human groups and societies (for a review, see Gelfand et al., 2024). Social norms constantly evolve through the dynamics of emergence, persistence and change (Gelfand et al., 2024). In recent years, emerging AI technologies have also become a disruptive force in the maintenance of social norms and pose new challenges in understanding what people believe is acceptable and what people likely do in social interactions with others. In particular, AI‐MCs have the potential to tip the normative balance by enabling people to engage in behaviour that is counter‐normative and shifting the blame on the AI systems (Dong & Bocian, 2024; von Schenk et al., 2023).

Theoretical accounts of social norms state that people expect others to be authentic and honest in interpersonal communications, which serve as important signals for trust (Jordan et al., 2017; Ohtsubo et al., 2010). In this context, using AI‐MCs may induce negative interpersonal judgements such as the character of untrustworthiness, given people's negative evaluations of AI‐generated content in general (Donath, 2007; Jakesch et al., 2019). People believe AI lacks particularly subjective capabilities required for conveying emotions and attributes that are essential and unique to humans (authenticity, empathy, morality, etc.; Bigman & Gray, 2018; Castelo et al., 2019; Gray et al., 2007; Morewedge, 2022). However, these previous studies often examined people's perceptions of AI systems in isolation, rather than their use as mediators in an interpersonal communicative context (Hancock et al., 2020).

We reason that people would evaluate AI negatively in human–human communication contexts and this negative evaluation would also depend on the transparency of AI‐MC uses. In AI‐MC contexts, the disclosure of AI‐produced advice influences people's perceived credibility and adherence (Böhm et al., 2023; Leib et al.,^2024^). For example, people accept identical advice for performance improvement less when the advice is labelled as AI‐rather than human‐generated (Tong et al., 2021). Since the disclosure of AI‐generated advice often yields negative reactions, in the context of interpersonal communications, people may see AI‐MCs more negatively when used secretly rather than publicly. Secret (vs. public) AI‐MC uses may be seen as deceptive and immoral by overclaiming the efforts, violating expectations of authenticity and honesty and falsely signalling trust‐related character (Celniker et al., 2023; Dong et al., 2021; Jordan et al., 2017). People may, therefore, see secret (vs. public) AI‐MC users as pursuing undeserved moral credits and also judge the users' character more negatively (Dong et al., 2021; Jordan et al., 2017).

Normative judgements also come in different forms, which can have nuanced effects on downstream behaviours. For example, beliefs about what most other people do (i.e., descriptive norms) can shape people's perceived acceptability of morally dubious behaviours (i.e., injunctive norms; Eriksson et al., 2015). However, what people believe as right and wrong does not always align with their actual behaviours (Köbis et al., 2022; Schwartz & Inbar, 2023) and people may impose different moral standards on themselves versus others (Valdesolo & DeSteno, 2007; Weiss et al., 2018). In particular, when it comes to potentially problematic AI uses, people's normative judgements about themselves (vs. others) are more aligned with a good reputation and positive self‐image (Dong & Bocian, 2024). Relatedly, people's moral acceptability judgements of others' (vs. their own) behaviours are often a better predictor of their actual behaviours (Perugini & Leone, 2009). To attenuate reputation concerns or social desirability, people are often asked to speculate most other people's thoughts and actions instead of directly reporting their feelings and behavioural intentions (Danioni & Barni, 2021; Dong et al., 2023).

In the current studies, we examine whether secret AI‐MCs are perceived as less acceptable than open AI‐MCs, whether people expect others to use AI‐MCs more than they would themselves and whether the relationship between perceptions of acceptability and expectations for use is impacted by the user in question (self vs. other) or by the nature of the AI‐MC (secret vs. open). Beyond these questions, we explore the roles of individual factors, including beliefs about and familiarity with AI‐MCs. In line with popular claims, we find evidence for the impact of transparency on acceptability but only when participants are directly contrasting these two versions of AI‐MCs. Further, in line with normative psychology theories, we find robust differences in attitudes towards one's own versus others' use.

## STUDY 1

### Method

#### Participants

Study 1 examined whether acceptability would be impacted by the *transparency* of the tool use (secret or open) and whether usage expectations would be impacted by the *user* in question (self or other). A representative sample of UK participants (*N* = 477) aged 18–75 (*M* = 48.48, SD = 15.83; females = 266^1^) stratified across age, sex and ethnicity was recruited through Prolific, a subject pool for online experiments (Palan & Schitter, 2018). Participants provided informed consent, indicated their demographics and read an introductory text on AI and communication. They then attended our online experiment.^2^ Data was collected in August 2021.

#### Study design

**FIGURE 1:**
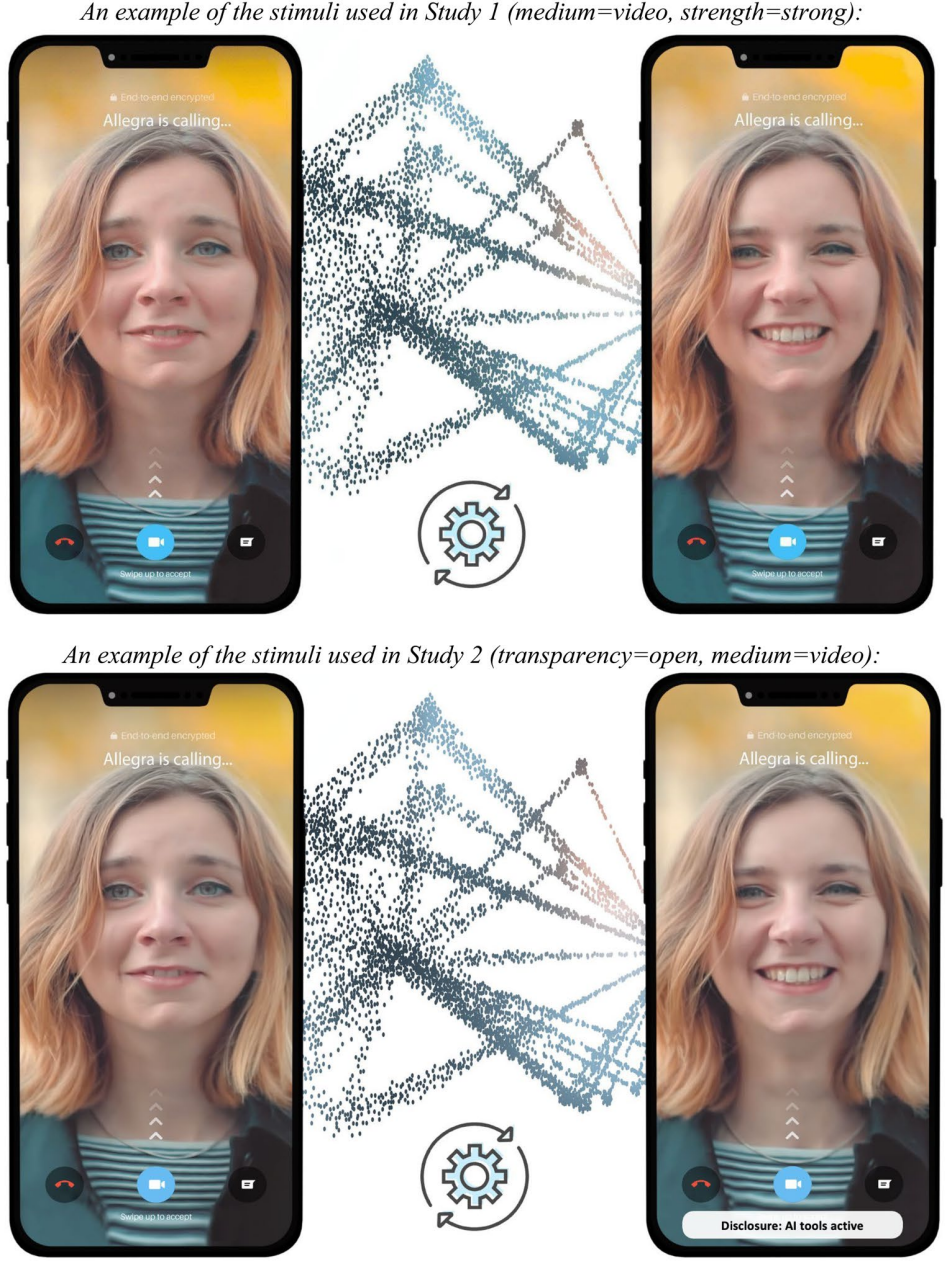
Examples of stimuli from Study 1 (top) and Study 2 (bottom). See OSF for more examples.

We employ a 2 (transparency: open vs. secret use) by 3 (medium: text vs. audio vs. video) by 2 (manipulation strength: weak vs. strong) within‐subjects design. Participants were presented with secret and open AI‐MCs and indicated expectations about their own versus others' AI‐MC use. All participants saw six examples of AI‐MCs: three mediums, at two augmentation strengths. The stimuli were created by the research team to show realistic scenarios of AI usage in communication, such as a video call that enhances appearance (weak) or facial expressions (strong video manipulation) or a writing assistant that improves writing style (weak) or content (strong textual manipulation). The scenarios were shown embedded in real‐world applications like FaceTime or Gmail and displayed the original content, an AI animation, as well as the content after AI manipulation (see Figure 1 and Data S1 for examples). The audio and video vignettes were shown as static images only. The full set of stimuli is included in the OSF repository. The study concluded by thanking participants for their time and effort.

For each stimulus shown, every participant was asked four questions, two about *acceptability* (1) How acceptable do you think it is for someone to openly use this technology? (2) How acceptable do you think it is for someone to secretly use this technology? [1 = Very unacceptable to 5 = Very acceptable] and two about *usage expectations* (1) How likely are others to use such technology? and (2) How likely are you to use such technology? [1 = Very unlikely to 5 = Very likely].

#### Statistical methods

To analyse our results, we employed linear mixed models predicting ‘acceptability’ and ‘usage expectations’ from the AI‐MC's transparency (secret or open) and user (self or other), respectively, using the lme4 package in R. In both models, we accounted for the nested data structure with random intercept effects for participant, medium and strengths. This also reflected our goal to investigate the relationships between user/transparency and AI‐MC usage expectations and acceptability, using different mediums and levels of modification strength to strengthen the robustness and generalisability of our results. Additionally, our rationale was reinforced by the rapidly evolving nature of AI technologies, necessitating a focus on overarching trends rather than specific tool attributes. Researchers interested in medium‐ or strength‐specific effects are invited to examine our data available at https://osf.io/ta5hu/. While Study 1 was an exploratory step, we explicitly pre‐registered our analytical approach for Study 2 (see below).

### RESULTS

#### Acceptability

**FIGURE 2:**
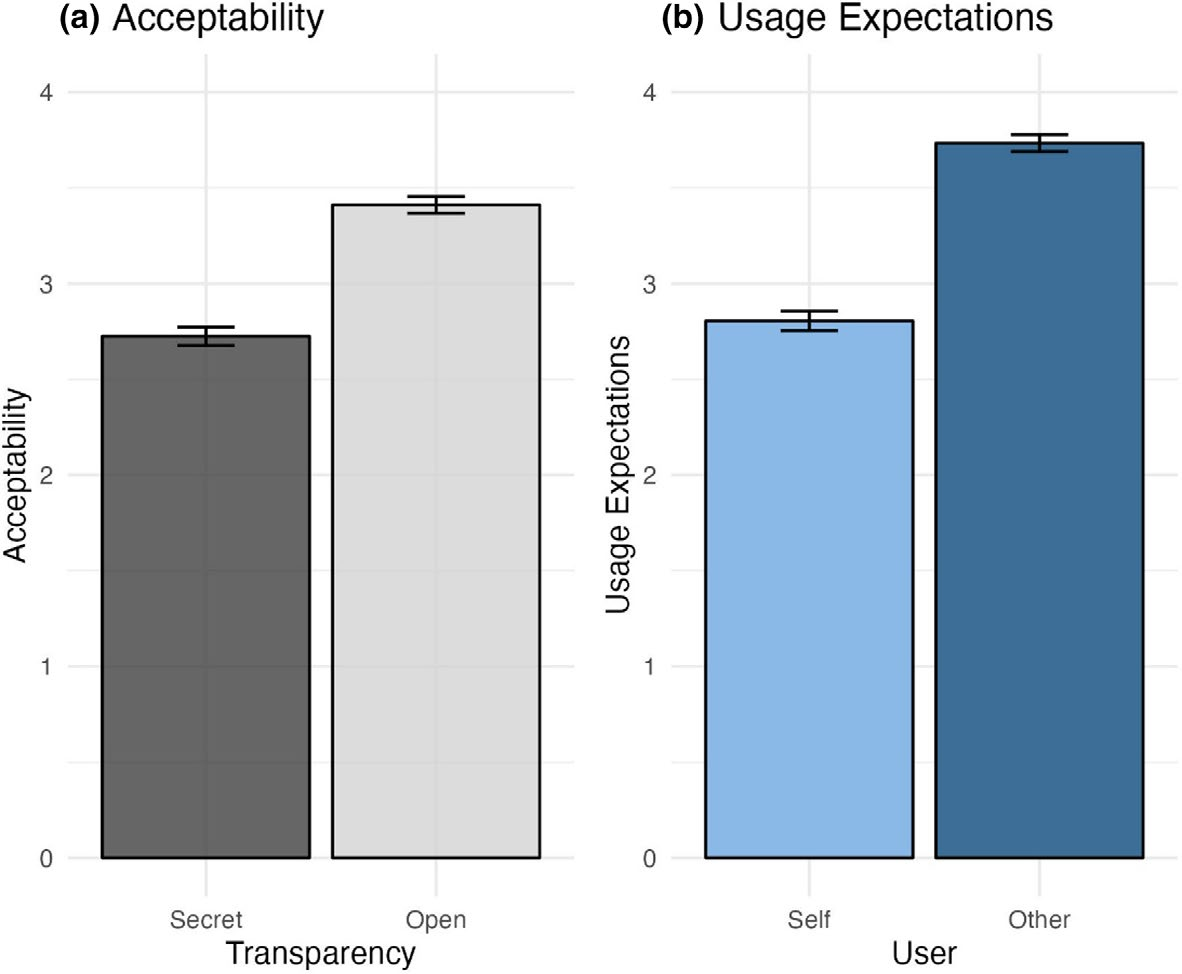
The results from Study 1 indicate that participants were more accepting of open than secret use of AI‐MCs (a) and that they expected others to use AI‐MCs more than they would themselves (b). Error bars are ± 1 SE.

Participants were less accepting of secret than open AI‐MC use (Figure 2), signified by a significant main effect of transparency (*B* = −0.69, 95% CI [−0.74, −0.64], *t*(5718) = −26.53, *p* < .001). Thus, people were less accepting of secret use of AI‐MCs (eM = 2.72, SE = .25) than open use (eM = 3.41, SE = .25).

#### Usage expectations

Participants believed others would use AI‐MCs more than they would themselves (Figure 2), signified by a significant main effect of the user (*B* = 0.93, 95% CI [0.88, 0.98], *t*(5718) = 33.98, *p* < .001). Thus, people expected that others (eM = 3.73, SE = .19) would use AI‐MCs more than they would themselves (eM = 2.80, SE = .19).

Study 1 found that participants were more accepting of open than secret AI‐MC use and that participants believed others were more likely to use AI‐MCs than they were themselves. This was a critical step to confirm, for the first time, that we are aware of, that people are explicitly less accepting of secret than open AI‐MCs. We were able to establish explicit beliefs by having participants directly contrast secret and open versions. However, by this within‐subjects design, Study 1 enhanced the salience of contrasts between secret versus open use and between use by self versus other. This, in turn, might have enhanced participants' sensitivity towards the contrasts in transparency (secret vs. open use) and user (self vs. other; Hsee, 1996; Hsee & Zhang, 2010). Thus, in Study 2, we varied transparency and user according to a between‐subjects design that allowed us to test whether our results were robust when the relevant contrasts were less salient.

## STUDY 2

Study 2 was pre‐registered through the OSF at https://osf.io/ta5hu/. Our main hypotheses, sample size, power analysis and all statistical analyses were pre‐registered. Our final sample size was slightly under the pre‐registered target sample size of 800 as 35 participants were excluded for incomplete data or failing attention checks. All pre‐registered analyses are described below while additional, exploratory analyses of the relationship between our main findings and key demographics – gender, age and education – are provided in Data S1.

Study 2 was a confirmatory follow‐up study in which we sought to pre‐register and replicate our findings from Study 1, in particular, that participants were less accepting of the secret than the open use of AI‐MCs and that they perceived others to be more likely to use AI‐MCs than themselves. Additionally, we also sought to assess the relationship between perceived acceptability and usage expectations for own versus others' AI‐MC use. As a further robustness check, we manipulated transparency and user between‐subjects and thereby reduced the salience of the relevant contrast in Study 2, ultimately preventing participants from making relative evaluations.

### METHOD

#### Participants

We recruited a representative sample of the US population through Prolific. The sample was stratified across age, sex and ethnicity (*N* = 765) and aged 18–93 (*M* = 45.47, SD = 15.75; females = 375^3^). We conducted a power analysis prior to the collection of data. The analysis used the results of Study 1 to estimate how many participants would be required to detect the effect size of interest ($\eta^{2}$ = 0.01) with sufficiently high probability at a significance level of α = 0.05, given the observed variance in the outcome variables of Study 1. The bootstrap estimates, included in the OSF repository, indicate an 80–82% probability of detecting relevant effects in both acceptability and likelihood of use for a sample of 800 respondents. Data was collected in August 2022.

#### Method

We implemented a 2 (transparency: open vs. secret; between‐subjects) by 2 (user: self vs. other; between‐subjects) by 3 (medium: text vs. voice vs. video; within‐subjects) experimental design. Participants provided informed consent, indicated their demographics and read a short introduction to AI. Each participant was allocated to one of four conditions such that they saw examples of AI‐MCs that were either open *or* secret, thus manipulating transparency. According to the other between‐subjects manipulation, participants evaluated AI‐MC use either by themselves *or* by others. As in Study 1, to test the robustness and generalisability of our results, participants saw three examples of AI‐MCs: text, voice and video. To limit the number of comparisons, only the ‘strong’ versions from Study 1 were used (see Figure 1).

For each of the three AI‐MCs, participants were asked to evaluate usage likelihood [‘How likely are you/others to use such technology?’ on a scale from 0 = Very Unlikely to 100 = Very Likely] and acceptability [‘How acceptable do you think it would be for you/others to use such technology?’ on a scale from 0 = Very Unacceptable to 100 = Very Acceptable]. For example, a participant in the ‘open‐self’ condition was presented with an AI‐MC (i.e., text, voice or video message) that was accompanied by a notification declaring the use of AI assistance tools and was then asked about whether they would use the AI‐MC and how acceptable it is to do so. Meanwhile, a participant in the ‘secret‐other’ condition was presented with an AI‐MC (i.e., text, voice or video message) that was not accompanied by a notification about the use of AI assistance tools, before being asked about whether others would use such an AI‐MC and how acceptable it would be for others to do so.

We explored several potentially related individual factors: (1) the respondent's familiarity with AI‐MCs (e.g., ‘How familiar are you with AI‐powered video editing tools that alter one's appearance during a video call? [0 = Not familiar at all to 100 = Extremely familiar]’), (2) whether participants believed AI‐MC use would lead to a loss of information (e.g., ‘How much information is lost if a person uses AI‐powered video editing tools to alter their appearance during a video call? [0 = None at all to 100 = A great deal]’) and (3) whether they perceived AI‐MC use or non‐use as indicative of another person's character (e.g., ‘Whether or not another person uses AI‐powered video editing tools tells me something about that other person's character. [0 = Strongly disagree to 100 = Strongly agree]’). These questions were asked separately for each AI‐MC medium and interspersed by two attention checks (‘Select 0/100 if you are paying attention.’). Participants who failed an attention check were excluded from the study.

#### Statistical method

As in Study 1, the data from Study 2 was analysed using linear mixed‐effects models predicting ‘acceptability’ and ‘usage expectations’ from the AI‐MC's transparency (secret or open) and user (self or other) using the lme4 package in R. In line with the nested structure of the data, we include ‘participant’ and ‘medium’ as random intercept effects. This approach was pre‐registered at the project's OSF page https://osf.io/ta5hu/. Similarly, when exploring the relationships between individual factors (e.g., familiarity) and our dependent variables, we used the same linear mixed models but with the addition of a fixed factor for the individual factor score. An additional exploration of the relationships between demographic factors and our key variables is included in Data S1.

### RESULTS

#### Acceptability

**FIGURE 3:**
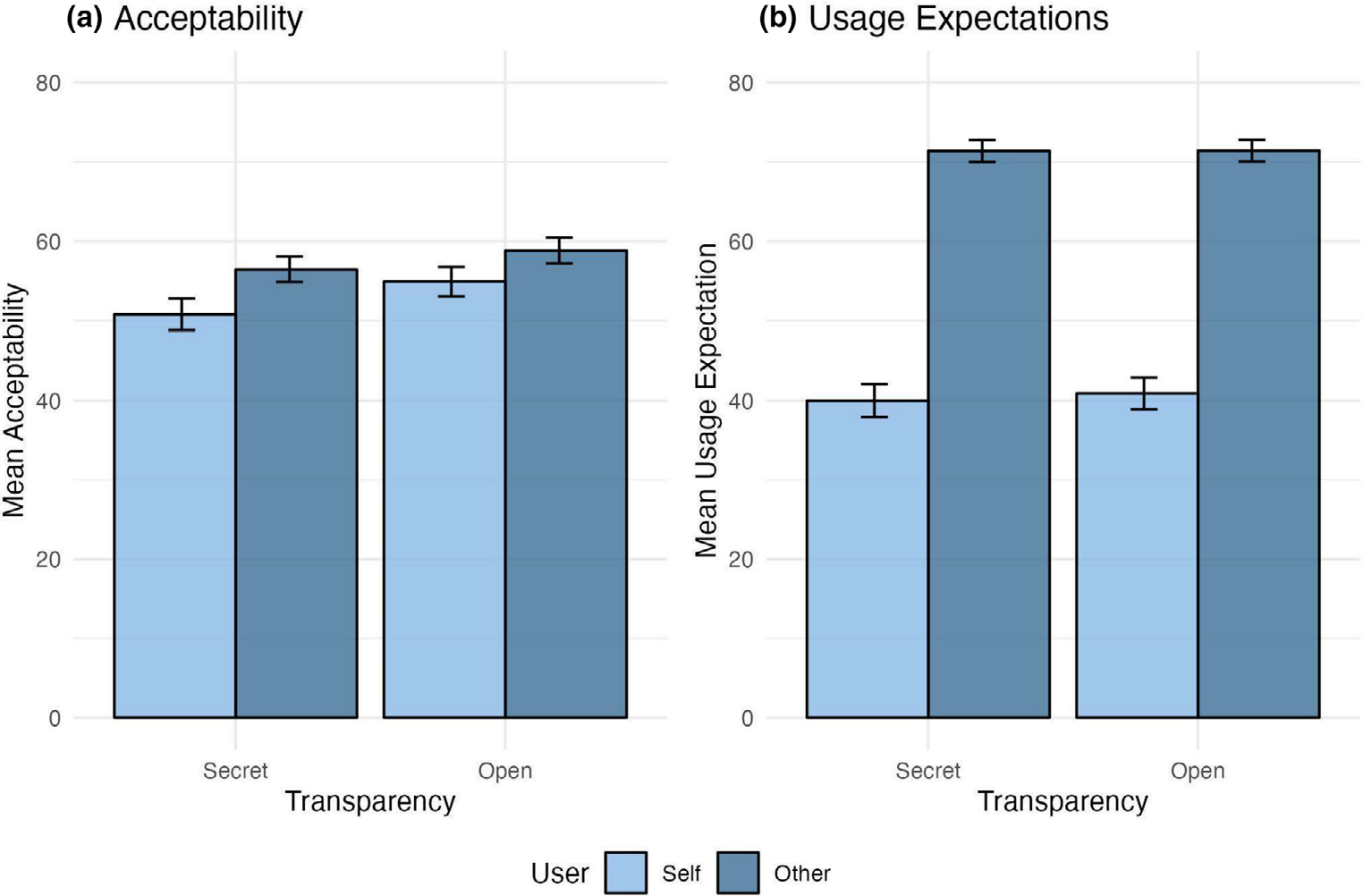
Acceptability ratings (a) did not differ between secret and open AI‐MCs or for own versus others' use. Usage expectations (b) did not differ for secret and open AI‐MCs; however, people expected others to use AI‐MC to a much greater extent than they would themselves. Error bars are ± 1 SE.

In Study 2, where the between‐subject manipulation of open vs. secret AI‐MC use prevented individual participants from making relative evaluations, acceptability ratings did not differ significantly for transparency, *B* = 3.25, 95% CI [−0.23, 6.73], *t*(2288) = 1.83, *p* = .067. At the descriptive level, there was still a difference whereby participants were more accepting of open (eM = 56.87, SE = 4.94) than secret AI‐MC use (eM = 53.62, SE = 4.94; see Figure 3a). Meanwhile, the main effect of user was significant (*B* = 4.79, 95% CI [1.31, 8.26], *t*(2288) = 2.70, *p* = .007), signifying that participants were less accepting when considering their own use (eM = 52.85, SE = 4.94) than that of others, eM = 57.64, SE = 4.94 (see Figure 3a). The interaction of transparency and user on acceptability was not significant (*B* = −1.74, 95% CI [−8.69, 5.22], *t*(2288) = −0.49, *p* = .624).

#### Usage expectations

Effects of transparency and user on usage expectations paralleled those on acceptability evaluations observed in Study 2. In particular, transparency did not affect usage expectations (*B* = 0.47, 95% CI [−2.92, 3.86], *t*(2288) = 0.27, *p* = .787), signifying that participants did not have different usage expectations for open (eM = 56.16, SE = 4.10) compared to secret AI‐MCs (eM = 55.70, SE = 4.10; see Figure 3b). Meanwhile, the main effect of the user was significant (*B* = 30.97, 95% CI [27.58, 34.36], *t*(2288) = 17.92, *p* < .001), indicating that participants expected others (eM = 71.41, SE = 4.10) to be more likely to use AI‐MCs than themselves (eM = 40.44, SE = 4.10; Figure 3b). The interaction between transparency and user was also not significant (*B* = −0.85, 95% CI [−7.63, 5.92], *t*(2288) = −0.25, *p* = .805).

#### Usage expectations and acceptability

To explore the relationship between acceptability and usage evaluations, we used a linear mixed effects model predicting rating scores from user, transparency and evaluation type (acceptability, usage expectations), as well as their two‐ and three‐way interactions. Participant and medium were included as random intercept effects. We found a significant two‐way interaction between user and evaluation (*B* = 26.18, 95% CI [23.62, 28.75], *t*(4579) = 20.00, *p* < .001). Additionally, we also observed a smaller two‐way interaction between transparency and evaluation (*B* = −2.78, 95% CI [−5.35, −0.22], *t*(4579) = −2.13, *p* = .034). These effects were not qualified by a three‐way interaction (*p* = .736; see Appendix for other effects in the model).

We explored the user‐by‐evaluation interaction by examining the relationship between acceptability and usage expectations separately for participants considering their *own* AI‐MC use (user = self) and for participants considering *others'* AI‐MC use (user = other). We used a linear mixed model predicting usage expectations from acceptability scores with participant and medium as random effects. The relationship between acceptability and usage expectations was stronger for ‘self’ AI‐MC use (*B* = 0.78, 95% CI [0.73, 0.82], *t*(1126) = 35.82, *p* < .001) than for ‘other’ AI‐MC use (*B* = 0.34, 95% CI [0.30, 0.38], *t*(1159) = 15.56, *p* < .001). This suggests that people may consider the acceptability of AI‐MCs to a greater extent when evaluating whether they expect to use AI‐MCs themselves than when evaluating whether they believe others will use AI‐MCs.

**FIGURE 4:**
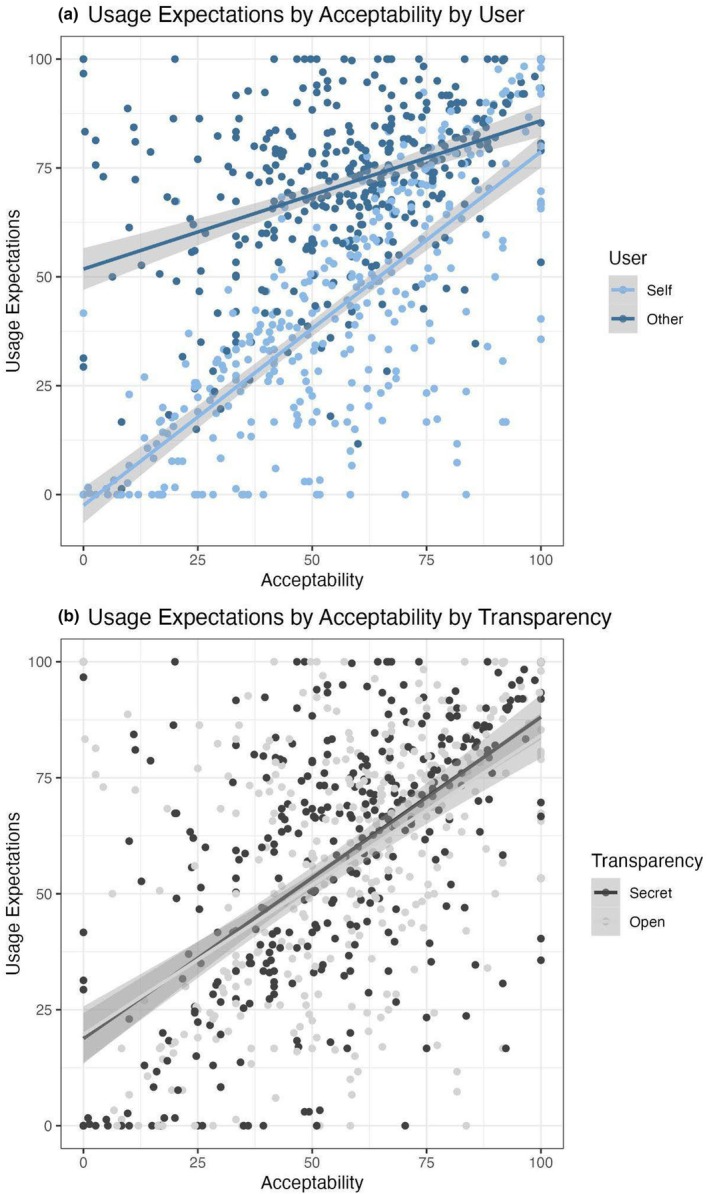
Panel a shows a stronger relationship between what is acceptable and expectations for use for participants evaluating their own use (light blue) than that of others (dark blue). Panel b shows similar relationships between acceptability and usage for secret and open AI‐MCs. NB: Points reflect mean usage and acceptability scores per participant, averaging across the medium. Lines of best fit (usage ~ acceptability) are included separately for self and other (a) and secret and open (b), with 95% confidence intervals shaded around each line.

Similarly, to explore the transparency‐by‐evaluation interaction, we examined the relationship between acceptability and usage expectations separately for participants considering open AI‐MCs and those considering secret AI‐MCs. We used a linear mixed model predicting usage expectations from acceptability scores with participant and medium as random effects. However, the relationship between acceptability and usage expectations was similar for open AI‐MCs (*B* = 0.59, 95% CI [0.54, 0.63], *t*(1129) = 23.81, *p* < .001) and secret AI‐MCs (*B* = 0.58, 95% CI [0.53, 0.63], *t*(1156) = 23.26, *p* < .001). This suggests that the relationship between acceptability and usage expectations remains consistent for both open and secret AI‐MCs (see Figure 4).

#### Individual factors

In addition to our primary analyses, we examined how several individual factors related to AI‐MC usage expectations and acceptability: familiarity with AI [Familiarity; *M* = 26.53, SD = 22.99], beliefs about whether AI‐MC use causes information loss [Information; *M* = 40.97, SD = 22.58] and beliefs about whether AI‐MC use is indicative of the user's character [Character; *M* = 57.88, SD = 23.90]. We ran several regression analyses using linear mixed models with usage or acceptability regressed on user, transparency and each of our individual factors (separately), with medium and participant included as random effects.

We found that greater acceptability was associated with greater familiarity with AI‐MCs (*B* = 0.15, *p* < .001), lower expectations of information loss due to AI‐MC use (*B* = −0.41, *p* < .001) and weaker character signal provided by AI‐MC use (*B* = −0.36, *p* < .001). Greater usage expectations were associated with greater familiarity (*B* = 0.19, *p* < .001), lower information loss (*B* = −0.23, *p* < .001) and lower character signals (*B* = −0.20, *p* < .001). For familiarity and character signals, the associations with usage expectations were stronger for participants considering their own potential AI‐MC use than that of others (see Appendix for extended results).

#### Summary

Study 2 robustly replicated that participants believed others were more likely to use AI‐MCs than they were themselves – even when participants were prevented from making relative judgements by the nature of the between‐subjects design. Meanwhile, the effect of transparency on acceptability was only marginally significant in Study 2, where the contrast between secret and open AI‐MC use was not salient. Participants rating open AI‐MCs were only slightly more accepting than those rating secret AI‐MCs.

Study 2 also explored the relationship between judgements of what is acceptable and expectations for use, indicating that judgements might be less aligned when people consider others' use than when they consider their own use. Additionally, our exploratory analyses of individual factors revealed that stronger familiarity, weaker beliefs that AI‐MC use causes information loss and lower beliefs that AI‐MC use is indicative of the user's character, predict greater perceived acceptability and higher usage expectations, especially when considering one's own use.

Our primary findings highlight that people are concerned about AI‐MC use, particularly, that of others and of secret AI‐MCs. However, the positive relationships between familiarity, acceptability and usage suggest that these concerns may ease as individuals become more accustomed to AI‐MCs.

## DISCUSSION

We are witnessing exponential growth in the development and uptake of AI‐MCs. One exemplar, ChatGPT, gained 1 million users in just 5 days (Buchholz, 2023). Despite this proliferation, little is known about individual outlooks on this development nor, consequently, about how we can expect this development to evolve. In this regard, we studied people's acceptability of and expectations for AI‐MC use and explored two key contextual factors: AI‐MC transparency (open vs. secret use) and the user in question (self vs. others). Both judgements of (1) what behaviours are acceptable or not (i.e., injunctive norms) and (2) what most other people typically do (i.e., descriptive norms) are crucial predictors of people's actual behaviours (e.g., Bicchieri, 2016; Bicchieri & Xiao, 2009). Moreover, public versus private and self versus others are often contextual factors that influence such normative judgements (e.g., Valdesolo & DeSteno, 2007; Vogt et al., 2016; Weiss et al., 2018).

In the current studies, people rated secret AI‐MCs – those that do not notify the receiver of AI involvement – as less acceptable than open AI‐MCs – those that did notify the receiver of the AI involvement. This effect was stronger in Study 1 when the questions about open and secret use were presented simultaneously. Notably, the effect was only marginally significant in Study 2, where open and secret AI‐MCs operation modes were not directly contrasted. These findings highlight that people, in principle, care about transparency in AI‐MC use. However, the acceptability of AI use will depend on how the technologies are introduced and how transparency modes are being communicated. These findings on transparency may not only have implications on consumer preference but also in the guidance for ethical AI development, particularly in attempts to avoid the secretive misuse of such tools (Chee, 2023).

We also observed robust self‐other discrepancies. People expected others to be more likely to use AI‐MCs than themselves (Studies 1 and 2). They also perceived others' potential AI‐MC use as more acceptable than their own use (Study 2). These findings are generally in line with previous research establishing important self‐other differences in AI‐attitudes (e.g., Purcell & Bonnefon, 2023a, 2023b) and those suggesting a mutually reinforcing relationship between descriptive norms of what people typically do and injunctive norms of what behaviours are deemed acceptable (Eriksson et al., 2015). However, it should be noted that when we asked participants to evaluate others, the descriptions were rather general without specifying identities (e.g., gender or political orientation) or relationships (e.g., friends or colleagues). Even though the self‐other distinctions remained regardless of whether we referred to others in singular (‘someone’ in Study 1) or plural (‘others’ in Study 2) terms, varied abstract versus concrete information about others may also influence people's acceptability and usage judgements and self‐other discrepancies (Lammers, 2012). Future research may test the robustness of self‐other discrepancies in AI‐MC evaluations with different operationalizations of others.

Importantly, although in Study 2 we observed a positive correlation between usage likelihood and acceptability judgement, this correlation was stronger in people's evaluation of themselves than others. Participants' expectations for their *own* use were strongly aligned with their perceptions of what was acceptable, whereas this was less so for their expectations of *others'* usage. It is possible that people expect their own AI‐MC use to be responsible in so far as it reflects what they deem as acceptable, they do not expect others' AI‐MC use to follow the same principles. Instead, they may believe that others' AI‐MC use can be complicated by contextual factors other than merely guided by intrinsic moral beliefs. These arguments are largely speculative since we do not have data on others' actual AI‐MC usage. However, we find conceptual support from previous literature on morals, which suggests that people often believe themselves to be both morally unique (Purcell & Bonnefon, 2023a, 2023b) and more moral than other people but such beliefs are often illusory and do not predict superior moral performances (Dong, 2023; Hoorens, 1993). Put differently, people may hold broadly negative outlooks for the evolution of AI‐MCs and AI‐MC use such that they believe uptake will be greater for others (Bicchieri, 2016; Köbis et al., 2022) and will be less aligned with their individual beliefs about what is acceptable. Though people's beliefs about others' (vs. their own) thoughts and actions are often more indicative of their actual behaviours (Danioni & Barni, 2021; Dong et al., 2023), future research should also collect benchmark data on people's actual use of AI‐MCs and test the relationship between acceptability judgement and actual usage.

Our exploration of individual factors gives some indication of the possible drivers of acceptability and usage expectations. We found that what people deem as more acceptable is related to their beliefs about whether AI‐MC use is indicative of the user's character, whether they believe AI‐MC use yields a loss of information and their familiarity with AI‐MCs. Sensibly, this suggests that as AI‐MCs become more mainstream and familiarity increases, so too will acceptability. Additionally, these findings may inform our interpretation of the self‐other distinctions that emerge across our primary claims. It appears that people's perceptions of whether AI‐MC use tells us something about the user's character are strongly related to ratings of one's own but not others' AI‐MC use. Indeed, when people believe AI‐MC use can be morally negative, they show harsher standards on themselves than others to manage their reputation (Dong et al., 2023); hence, the lower reported expectations for their own use could reflect a strategic move for reputation management.

In regard to AI‐MCs, the current studies support the notions that transparency and user are associated with judgements of what is acceptable and expectations of usage. We believe these factors carry pragmatic and theoretical relevance across all AI‐MCs. However, there are undoubtedly many other factors that will affect acceptability and usage expectations in specific contexts. For example, the relationship between communicators (e.g., manager–employee, parent–child, business–customer), the subject of the communication (e.g., policy, schoolwork, apology) or the outcomes of the communication (e.g., school grades, voting behaviour, phishing) are likely to influence the acceptability of specific AI communication technologies. Indeed, other research has examined how the use of AI‐MCs affects people's perception of authenticity at the workplace (Glikson & Asscher, 2023) and user‐engagement on TikTok (Kang & Lou, 2022). More work testing our findings in different contexts, for example, personal, work and education contexts, would be a fruitful avenue for future research.

Recently, the European Union created the Artificial Intelligence Act (AI‐ACT) seeking to govern and regulate AI technologies (Artificial Intelligence Act, 2024), using a risk‐based approach. As outlined in the introduction, AI‐MCs can be used to manipulate and defraud people, therefore posing a potentially growing risk. The AI‐ACT also emphasizes that the implementation of AI systems needs to uphold fundamental rights, ensure user safety and importantly promote transparency around their use. Our research characterizes people's demand for such transparency, hence informing the development, deployment and societal acceptance of AI‐MCs. In that way, our research aligns with AI‐ACT's emphasis on human‐centric and trustworthy AI, highlighting the increasing relevance of regulatory environments in shaping technology's societal integration.

## AUTHOR CONTRIBUTIONS

**Zoe A. Purcell:** Conceptualization; methodology; software; data curation; investigation; validation; formal analysis; visualization; project administration; resources; writing – original draft; writing – review and editing; funding acquisition. **Mengchen Dong:** Conceptualization; methodology; funding acquisition; writing – original draft; writing – review and editing. **Anne‐Marie Nussberger:** Conceptualization; methodology; formal analysis; funding acquisition; writing – review and editing. **Nils Köbis:** Conceptualization; methodology; funding acquisition; writing – review and editing. **Maurice Jakesch:** Conceptualization; methodology; writing – review and editing; visualization.

## CONFLICT OF INTEREST STATEMENT

No conflicts of interest.

## Supporting information


Data S1.

## Data Availability

The data that support the findings of this study and the experiment stimuli are openly available in the Open Science Framework, https://osf.io/ta5hu/.

## Footnotes

^1^
Other genders: Male = 200, non‐binary = 4, prefer not to say = 3, no response = 4.

^2^
This experiment was part of an omnibus survey, the sample size was determined by other, confirmatory studies.

^3^
Other genders: Male = 379, non‐binary = 8, prefer not to say = 3.

## EXTENDED RESULTS FOR STUDY 2

### Acceptability and usage expectations

To examine the relationship between ratings of acceptability and usage expectations and their interactions with transparency and user, we used a linear mixed model predicting rating scores from user, transparency and ‘evaluation’ (acceptability or usage expectations). Participant and medium were included as random effects. The model's intercept, corresponding to the model mean, is at 55.59 (95% CI [47.35, 63.83], *t*(4579) = 13.23, *p* < .001). Within this model, the effect of transparency was statistically non‐significant and positive (*B* = 1.86, 95% CI [−1.24, 4.96], *t*(4579) = 1.18, *p* = .239). The effect of the user was statistically significant and positive (*B* = 17.88, 95% CI [14.78, 20.98], *t*(4579) = 11.31, *p* < .001). The effect of the evaluation was statistically non‐significant and positive (*B* = 0.68, 95% CI [−0.60, 1.97], *t*(4579) = 1.04, *p* = .297). The effect of the transparency × user interaction was statistically non‐significant and negative (*B* = −1.30, 95% CI [−7.49, 4.90], *t*(4579) = −0.41, *p* = .682). The interaction effect of transparency × evaluation was statistically significant and negative (*B* = −2.78, 95% CI [−5.35, −0.22], *t*(4579) = −2.13, *p* = .034); see main text for more details. The effect of the user × evaluation interaction was statistically significant and positive (*B* = 26.18, 95% CI [23.62, 28.75], *t*(4579) = 20.00, *p* < .001); see main text for more details. The three‐way interaction effect of transparency × user × evaluation was statistically non‐significant and positive (*B* = 0.88, 95% CI [−4.25, 6.02], *t*(4579) = 0.34, *p* = .736).

### Individual factors

In addition to our primary analyses, we examined how three factors – familiarity, information loss and character signal – impacted the acceptability of AI‐MCs and people's usage expectations. Descriptive results are in Table A1; however, simple correlations should be interpreted with caution as linear modelling showed some interaction effects (see below and the main text).

**TABLE A1:** Descriptive statistics for Study 2: Overall means, standard deviations and paired‐sample correlations between key variables.

Variable	Mean (SD)	Paired sample Pearson R (*p*‐value)				
		1.	2.	3.	4.	5.
1. Use	56.15 (28.44)	1				
2. Acceptability	55.26 (24.64)	0.58 (<.001)	1			
3. Familiarity	26.53 (22.99)	0.19 (<.001)	0.17 (<.001)	1		
4. Information Loss	40.97 (22.58)	−0.23 (<.001)	−0.45 (<.001)	0.06 (.075)	1	
5. Character	57.88 (23.90)	−0.04 (.272)	−0.27 (<.001)	−.01 (.848)	0.34 (<.001)	1

*Note*: NB. Correlations should be interpreted with caution due to interactions with other factors (see below).

### Acceptability and familiarity

With the intercept at the mean: the effect of transparency was statistically non‐significant (*B* = 2.88, 95% CI [−1.19, 6.95], *t*(2284) = 1.39, *p* = .165); the effect of user was statistically significant and positive (*B* = 5.79, 95% CI [1.72, 9.86], *t*(2284) = 2.79, *p* = .005); the effect of familiarity was statistically significant and positive (*B* = 0.15, 95% CI [0.11, 0.20], *t*(2284) = 6.94, *p* < .001); the interaction effect of user by transparency was statistically non‐significant (*B* = 0.80, 95% CI [−7.34, 8.94], *t*(2284) = 0.19, *p* = .847); the interaction effect of familiarity by transparency was statistically non‐significant (*B* = 8.96e‐03, 95% CI [−0.07, 0.09], *t*(2284) = 0.21, *p* = 0.832); the interaction effect of familiarity by user is statistically non‐significant (*B* = −0.04, 95% CI [−0.12, 0.04], *t*(2284) = −0.92, *p* = 0.356); the interaction effect of familiarity by (transparency * user) was statistically non‐significant (*B* = −0.09, 95% CI [−0.26, 0.07], *t*(2284) = −1.10, *p* = 0.271).

### Acceptability and information loss

We employed a linear mixed model predicting ‘acceptability’ from the user (self, other), transparency (open, secret) and information loss [0–100] and all possible interactions between the three predictors. Participant and medium (text voice, video) were included as random effects. With the intercept at the mean: the effect of transparency was statistically non‐significant (*B* = −1.59, 95% CI [−6.16, 2.98], *t*(2284) = −0.68, *p* = .496); the effect of user was statistically non‐significant (*B* = 4.52, 95% CI [−0.05, 9.09], *t*(2284) = 1.94, *p* = .053); the effect of information loss was statistically significant and negative (*B* = −0.41, 95% CI [−0.46, −0.37], *t*(2284) = −19.03, *p* < .001); the interaction effect of user by transparency was statistically non‐significant (*B* = −3.64, 95% CI [−12.78, 5.50], *t*(2284) = −0.78, *p* = .435); the interaction effect of information loss by transparency is statistically significant and positive (*B* = 0.11, 95% CI [0.03, 0.19], *t*(2284) = 2.57, *p* = .010); the interaction effect of information loss by user is statistically non‐significant and negative (*B* = −2.62e‐03, 95% CI [−0.08, 0.08], *t*(2284) = −0.06, *p* = .950); the interaction effect of information loss by (transparency * user) is statistically non‐significant and positive (*B* = 0.07, 95% CI [−0.10, 0.23], *t*(2284) = 0.79, *p* = .429).

To explore the significant interaction between transparency and information loss, we ran a linear mixed model predicting acceptability from information loss with participant and medium as random effects. We then examined secret AI‐MCs separately to open AI‐MCs; the effect of information loss was stronger for secret than open AI‐MCs. For secret AI‐MCs, the effect of information loss was statistically significant and negative (*B* = −0.47, 95% CI [−0.53, −0.41], *t*(1156) = −15.73, *p* < .001). For open AI‐MCs, the effect of information loss was statistically significant and negative (*B* = −0.37, 95% CI [−0.43, −0.30], *t*(1129) = −11.46, *p* < .001). This suggests that as perceived information loss increases, acceptability decreases and that this relationship is stronger for secret than open AI‐MCs.

### Acceptability and character signal

We employed a linear mixed model predicting ‘usage’ from the user (self, other), transparency (open, secret) and character signal [0–100] and all possible interactions between the three predictors. Participant and medium (text voice, video) were included as random effects. With the intercept at the mean: the effect of transparency was statistically non‐significant (*B* = 3.13, 95% CI [−3.25, 9.51], *t*(2284) = 0.96, *p* = .336); the effect of user was statistically non‐significant (*B* = 4.46, 95% CI [−1.92, 10.83], *t*(2284) = 1.37, *p* = .171); the effect of character signal was statistically significant and negative (*B* = −0.36, 95% CI [−0.41, −0.31], *t*(2284) = −14.36, *p* < .001); the interaction effect of user by transparency was statistically non‐significant (*B* = −9.51, 95% CI [−22.26, 3.25], *t*(2284) = −1.46, *p* = .144); the interaction effect of character by transparency was statistically non‐significant (*B* = −9.96e‐04, 95% CI [−0.09, 0.09], *t*(2284) = −0.02, *p* = .983); the interaction effect of character by agent was statistically non‐significant (*B* = 0.02, 95% CI [−0.07, 0.12], *t*(2284) = 0.45, *p* = 0.650); the interaction effect of character by (transparency * agent) was statistically non‐significant (*B* = 0.13, 95% CI [−0.06, 0.32], *t*(2284) = 1.37, *p* = .171).

### Usage expectations and familiarity

We employed a linear mixed model predicting ‘usage’ from the user (self, other), transparency (open, secret) and familiarity [0–100] and all possible interactions between the three predictors. Participant and medium (text voice, video) were included as random effects. With the model's intercept at the mean: the effect of transparency was statistically non‐significant (*B* = 0.89, 95% CI [−3.02, 4.80], *t*(2284) = 0.45, *p* = .656); the effect of user was statistically significant and positive (*B* = 34.71, 95% CI [30.80, 38.62], *t*(2284) = 17.40, *p* < .001); the effect of familiarity was statistically significant and positive (*B* = 0.19, 95% CI [0.15, 0.23], *t*(2284) = 8.73, *p* < .001); the interaction effect of user by transparency was statistically non‐significant (*B* = 3.25, 95% CI [−4.57, 11.07], *t*(2284) = 0.82, *p* = .415); the interaction effect of familiarity by transparency was statistically non‐significant (*B* = −0.02, 95% CI [−0.10, 0.06], *t*(2284) = −0.54, *p* = .590); the interaction effect of familiarity by user was statistically significant and negative (*B* = −0.14, 95% CI [−0.22, −0.06], *t*(2284) = −3.49, *p* < .001); the interaction effect of familiarity on (transparency * user) was statistically non‐significant (*B* = −0.15, 95% CI [−0.31, 0.01], *t*(2284) = −1.82, *p* = .070).

To explore the interaction between familiarity and agent, we examined the effect of familiarity on usage for self and other separately. The effect of familiarity on usage was stronger for self than for other: For self, the effect of familiarity is statistically significant and positive (*B* = 0.22, 95% CI [0.15, 0.29], *t*(1126) = 6.26, *p* < .001) and for other, the effect of familiarity is statistically significant and positive (*B* = 0.13, 95% CI [0.09, 0.18], *t*(1159) = 5.49, *p* < .001). This suggests that as familiarity increased, so too did usage expectations and that this effect was stronger for people assessing AI‐MCs for their own use.

### Usage expectations and information loss

We employed a linear mixed model predicting ‘usage’ from the user (self, other), transparency (open, secret) and information loss [0–100] and all possible interactions between the three predictors. Participant and medium (text voice, video) were included as random effects. With the model's intercept at the mean: the effect of transparency was statistically non‐significant and negative (*B* = −1.97, 95% CI [−6.68, 2.75], *t*(2284) = −0.82, *p* = .414); the effect of user was statistically significant and positive (*B* = 19.67, 95% CI [14.96, 24.39], *t*(2284) = 8.18, *p* < .001); the effect of information loss was statistically significant and negative (*B* = −0.23, 95% CI [−0.27, −0.18], *t*(2284) = −10.28, *p* < .001); the interaction effect of user by transparency is statistically non‐significant and positive (*B* = 1.90, 95% CI [−7.53, 11.33], *t*(2284) = 0.39, *p* = .693); the interaction effect of information loss by transparency was statistically non‐significant and positive (*B* = 0.05, 95% CI [−0.03, 0.13], *t*(2284) = 1.19, *p* = .235); the interaction effect of information loss by user was statistically significant and positive (*B* = 0.27, 95% CI [0.19, 0.35], *t*(2284) = 6.40, *p* < .001); the interaction effect of information loss by (transparency * user) was statistically non‐significant and negative (*B* = −0.05, 95% CI [−0.22, 0.12], *t*(2284) = −0.58, *p* = .559).

To explore the interaction between information loss and user, we examined the effect of information loss on usage for self and other separately. The effect of information loss on usage was stronger for self than for other: For self, the effect of information loss is statistically significant and negative (*B* = −0.32, 95% CI [−0.39, −0.25], *t*(1126) = −9.08, *p* < .001) and similarly for other, the effect of information loss is statistically significant and negative (*B* = −0.14, 95% CI [−0.20, −0.09], *t*(1159) = −5.53, *p* < .001). Similar to the findings above, this suggests that as perceptions that AI‐MCs lead to information loss increase, usage expectations decrease and that this effect was strongest for participants considering AI‐MCs their own AI‐MC usage.

### Usage expectations and character signal

We employed a linear mixed model predicting ‘usage’ from the user (self, other), transparency (open, secret) and character signal [0–100] and all possible interactions between the three predictors. Participant and medium (text voice, video) were included as random effects. With the model's intercept at the mean: the effect of transparency was statistically non‐significant (*B* = 5.48, 95% CI [−0.86, 11.83], *t*(2284) = 1.69, *p* = .090); the effect of user was statistically significant and positive (*B* = 9.84, 95% CI [3.49, 16.18], *t*(2284) = 3.04, *p* = .002); the effect of character was statistically significant and negative (*B* = −0.20, 95% CI [−0.25, −0.16], *t*(2284) = −8.26, *p* < .001); the interaction effect of user by transparency was statistically non‐significant (*B* = −3.56, 95% CI [−16.25, 9.13], *t*(2284) = −0.55, *p* = .582); the interaction effect of character by transparency was statistically non‐significant (*B* = −0.09, 95% CI [−0.18, 5.53e‐03], *t*(2284) = −1.84, *p* = .065); the interaction effect of character by user was statistically significant and positive (*B* = 0.37, 95% CI [0.28, 0.47], *t*(2284) = 7.90, *p* < .001); the interaction effect of character on (transparency * user) was statistically non‐significant (*B* = 0.05, 95% CI [−0.14, 0.24], *t*(2284) = 0.53, *p* = 0.594).

To explore the interaction between character and user, we examined the effect of character on usage for self and other separately. The effect of character on usage was stronger for self than for other: For self, the effect of character is statistically significant and negative (*B* = −0.34, 95% CI [−0.42, −0.27], *t*(1126) = −8.61, *p* < .001) and for other, the effect of character is statistically significant and negative (*B* = −0.06, 95% CI [−0.12, −5.09e‐03], *t*(1159) = −2.14, *p* = .033). This suggests that as perceptions that AI‐MCs were indicative of one's character increased, usage expectations decreased and that this effect was stronger for participants considering their own use than those considering that of others.

## References

[bjop12727-bib-0001] Artificial Intelligence Act . (2024). European parliament . https://www.europarl.europa.eu/doceo/document/TA‐9‐2024‐0138_EN.pdf (Accessed: 12 April 2024)

[bjop12727-bib-0002] Bicchieri, C. (2016). Norms in the wild: How to diagnose, measure, and change social norms. Oxford University Press.

[bjop12727-bib-0003] Bicchieri, C. , & Xiao, E. (2009). Do the right thing: But only if others do so. Journal of Behavioral Decision Making, 22(2), 191–208. 10.1002/bdm.621

[bjop12727-bib-0004] Bigman, Y. E. , & Gray, K. (2018). People are averse to machines making moral decisions. Cognition, 181, 21–34. 10.1016/j.cognition.2018.08.003 30107256

[bjop12727-bib-0005] Böhm, R. , Jörling, M. , Reiter, L. , & Fuchs, C. (2023). People devalue generative AI's competence but not its advice in addressing societal and personal challenges. Communications Psychology, 1(1), 32. 10.1038/s44271-023-00032-x 39242905 PMC11332189

[bjop12727-bib-0006] Bonnefon, J.‐F. , Rahwan, I. , & Shariff, A. (2024). The moral psychology of artificial intelligence. Annual Review of Psychology, 75, 653–675. 10.1146/annurev-psych-030123-113559 37722750

[bjop12727-bib-0007] Browne, R. (2023). Italy became the first Western country to ban ChatGPT. Here's what other countries are doing. CNBC. https://www.cnbc.com/2023/04/04/italy‐has‐banned‐chatgpt‐heres‐what‐other‐countries‐are‐doing.html

[bjop12727-bib-0008] Buchholz, K. (2023). ChatGPT sprints to one million users. Statista infographics . https://www.statista.com/chart/29174/time‐to‐one‐million‐users

[bjop12727-bib-0009] Castelo, N. , Bos, M. W. , & Lehmann, D. R. (2019). Task‐dependent algorithm aversion. Journal of Marketing Research, 56(5), 809–825. 10.1177/0022243719851788

[bjop12727-bib-0010] Celniker, J. B. , Gregory, A. , Koo, H. J. , Piff, P. K. , Ditto, P. H. , & Shariff, A. F. (2023). The moralization of effort. Journal of Experimental Psychology: General, 152(1), 60–79. 10.1037/xge0001259 35901413

[bjop12727-bib-0011] Chee, F. Y. (2023). Europol sounds alarm about criminal use of ChatGPT, sees grim outlook. *Reuters* . https://www.reuters.com/technology/europol‐sounds‐alarm‐about‐criminal‐use‐chatgpt‐sees‐grim‐outlook‐2023‐03‐27/

[bjop12727-bib-0012] Chesney, B. , & Citron, D. (2019). Deep fakes: A looming challenge for privacy, democracy, and National Security. California Law Review, 107(6), 1753–1820.

[bjop12727-bib-0013] Danioni, F. , & Barni, D. (2021). Value priorities, impression management and self‐deceptive enhancement: Once again, much substance and a little bit of style. The Journal of Social Psychology, 161(2), 146–159. 10.1080/00224545.2020.1778619 32538711

[bjop12727-bib-0014] Donath, J. (2007). Signals in social supernets. Journal of Computer‐Mediated Communication, 13(1), 231–251. 10.1111/j.1083-6101.2007.00394.x

[bjop12727-bib-0015] Dong, M. (2023). False moral superiority and heroism. In Encyclopedia of heroism studies. Springer International Publishing.

[bjop12727-bib-0016] Dong, M. , & Bocian, B. (2024). Responsibility gaps and self‐interest bias: People attribute moral responsibility to AI for their own but not others' transgressions. Journal of Experimental Social Psychology, 111, 104584. 10.1016/j.jesp.2023.104584

[bjop12727-bib-0017] Dong, M. , Kupfer, T. R. , Yuan, S. , & van Prooijen, J.‐W. (2023). Being good to look good: Self‐reported moral character predicts moral double standards among reputation‐seeking individuals. British Journal of Psychology, 114(1), 244–261. 10.1111/bjop.12608 36330995 PMC10098708

[bjop12727-bib-0018] Dong, M. , van Prooijen, J. W. , & van Lange, P. A. (2021). Calculating hypocrites effect: Moral judgments of word‐deed contradictory transgressions depend on targets' competence. Journal of Theoretical Social Psychology, 5(4), 489–501. 10.1002/jts5.113

[bjop12727-bib-0019] Eriksson, K. , Strimling, P. , & Coultas, J. C. (2015). Bidirectional associations between descriptive and injunctive norms. Organizational Behavior and Human Decision Processes, 129, 59–69. 10.1016/j.obhdp.2014.09.011

[bjop12727-bib-0020] European Commission . (2024). Living guidelines on the responsible use of generative AI in research (First ed.). European Union. https://research‐and‐innovation.ec.europa.eu/document/download/2b6cf7e5‐36ac‐41cb‐aab5‐0d32050143dc_en?filename=ec_rtd_ai‐guidelines.pdf

[bjop12727-bib-0021] Gelfand, M. J. , Gavrilets, S. , & Nunn, N. (2024). Norm dynamics: Interdisciplinary perspectives on social norm emergence, persistence, and change. Annual Review of Psychology, 75, 341–378. 10.1146/annurev-psych-033020-013319 37906949

[bjop12727-bib-0600] Glikson, E. , & Asscher, O. (2023). AI‐mediated apology in a multilingual work context: Implications for perceived authenticity and willingness to forgive. Computers in Human Behavior, 140, 107592. 10.1016/j.chb.2022.107592

[bjop12727-bib-0022] Gray, H. M. , Gray, K. , & Wegner, D. M. (2007). Dimensions of mind perception. Science, 315(5812), 619. 10.1126/science.1134475 17272713

[bjop12727-bib-0023] Grueter, C. C. , & White, D. R. (2014). On the emergence of large‐scale human social integration and its antecedents in primates. Structure and Dynamics, 7(1). 10.5070/SD971024020

[bjop12727-bib-0024] Hancock, J. T. , Naaman, M. , & Levy, K. (2020). AI‐mediated communication: Definition, research agenda, and ethical considerations. Journal of Computer‐Mediated Communication, 25(1), 89–100. 10.1093/jcmc/zmz022

[bjop12727-bib-0025] Hoorens, V. (1993). Self‐enhancement and superiority biases in social comparison. European Review of Social Psychology, 4(1), 113–139. 10.1080/14792779343000040

[bjop12727-bib-0026] Hruschka, D. J. (2010). Friendship: Development, ecology, and evolution of a relationship (Vol. 5). Univ of California Press.

[bjop12727-bib-0027] Hsee, C. (1996). The evaluability hypothesis: An explanation for preference reversals between joint and separate evaluations of alternatives. Organizational Behavior and Human Decision Processes, 67(3), 247–257. 10.1006/obhd.1996.0077

[bjop12727-bib-0028] Hsee, C. K. , & Zhang, J. (2010). General evaluability theory. Perspectives on Psychological Science, 5(4), 343–355. 10.1177/1745691610374586 26162182

[bjop12727-bib-0029] Hsu, T. (2023). As Deepfakes Flourish, countries struggle with response. *The New York Times* . https://www.nytimes.com/2023/01/22/business/media/deepfake‐regulation‐difficulty.html

[bjop12727-bib-0030] Jago, A. S. (2019). Algorithms and authenticity. Academy of Management Discoveries, 5(1), 38–56. 10.5465/amd.2017.0002

[bjop12727-bib-0031] Jakesch, M. , Bhat, A. , Buschek, D. , Zalmanson, L. , & Naaman, M. (2023). Co‐writing with opinionated language models affects Users' views. *Proceedings of the 2023 CHI conference on human factors in computing systems*, 1–12.

[bjop12727-bib-0032] Jakesch, M. , French, M. , Ma, X. , Hancock, J. T. , & Naaman, M. (2019). AI‐mediated communication: How the perception that profile text was written by AI affects trustworthiness. *Proceedings of the 2019 CHI conference on human factors in computing systems*, 1–13.

[bjop12727-bib-0033] Jakesch, M. , Hancock, J. T. , & Naaman, M. (2023). Human heuristics for AI‐generated language are flawed. Proceedings of the National Academy of Sciences, 120(11), e2208839120. 10.1073/pnas.2208839120 PMC10089155 36881628

[bjop12727-bib-0034] Jordan, J. J. , Sommers, R. , Bloom, P. , & Rand, D. G. (2017). Why do we hate hypocrites? Evidence for a theory of false signaling. Psychological Science, 28(3), 356–368. 10.1177/0956797616685771 28107103

[bjop12727-bib-0035] Kang, H. , & Lou, C. (2022). AI agency vs. human agency: Understanding human–AI interactions on TikTok and their implications for user engagement. Journal of Computer‐Mediated Communication, 27(5), 1–13. 10.1093/jcmc/zmac014

[bjop12727-bib-0036] Köbis, N. , Bonnefon, J.‐F. , & Rahwan, I. (2021). Bad machines corrupt good morals. Nature Human Behaviour, 5(6), 679–685. 10.1038/s41562-021-01128-2 34083752

[bjop12727-bib-0037] Köbis, N. C. , Doležalová, B. , & Soraperra, I. (2022). Fooled twice: People cannot detect deepfakes but think they can. iScience, 24(11), 103364. 10.1016/j.isci.2021.103364 PMC8602050 34820608

[bjop12727-bib-0038] Lammers, J. (2012). Abstraction increases hypocrisy. Journal of Experimental Social Psychology, 48(2), 475–480. 10.1016/j.jesp.2011.07.006

[bjop12727-bib-0039] Leib, M. , Köbis, N. , Rilke, R. M. , Hagens, M. , & Irlenbusch, B. (2024). Corrupted by algorithms? How ai‐generated and human‐written advice shape (dis) honesty. The Economic Journal, 134(658), 766–784. 10.1093/ej/uead056

[bjop12727-bib-0040] Morewedge, C. K. (2022). Preference for human, not algorithm aversion. Trends in Cognitive Sciences, 26(10), 824–826. 10.1016/j.tics.2022.07.007 35941064

[bjop12727-bib-0041] Ohtsubo, Y. , Masuda, F. , Watanabe, E. , & Masuchi, A. (2010). Dishonesty invites costly third‐party punishment. Evolution and Human Behavior, 31(4), 259–264. 10.1016/j.evolhumbehav.2009.12.007

[bjop12727-bib-0042] Palan, S. , & Schitter, C. (2018). Prolific. ac—A subject pool for online experiments. Journal of Behavioral and Experimental Finance, 17, 22–27.

[bjop12727-bib-0043] Perugini, M. , & Leone, L. (2009). Implicit self‐concept and moral action. Journal of Research in Personality, 43(5), 747–754. 10.1016/j.jrp.2009.03.015

[bjop12727-bib-0044] Purcell, Z. A. , & Bonnefon, J.‐F. (2023a). Humans feel too special for machines to score their morals. PNAS Nexus, 2(6), pgad179. 10.1093/pnasnexus/pgad179 37325024 PMC10266524

[bjop12727-bib-0045] Purcell, Z. A. , & Bonnefon, J.‐F. (2023b). Research on artificial intelligence is reshaping our definition of morality. Psychological Inquiry, 34(2), 100–101. 10.1080/1047840X.2023.2248857

[bjop12727-bib-0046] Ruggeri, A. (2023). The problems with TikTok's controversial “beauty filters” . https://www.bbc.com/future/article/20230301‐the‐problems‐with‐tiktoks‐controversial‐beauty‐filters

[bjop12727-bib-0047] Schwartz, S. A. , & Inbar, Y. (2023). Is it good to feel bad about littering? Conflict between moral beliefs and behaviors for everyday transgressions. Cognition, 236, 105437. 36989917 10.1016/j.cognition.2023.105437

[bjop12727-bib-0048] Sundar, S. S. (2020). Rise of machine agency: A framework for studying the psychology of human–AI interaction (HAII). Journal of Computer‐Mediated Communication, 25(1), 74–88. 10.1093/jcmc/zmz026

[bjop12727-bib-0049] Sundar, S. S. , & Lee, E.‐J. (2022). Rethinking communication in the era of artificial intelligence. Human Communication Research, 48(3), 379–385. 10.1093/hcr/hqac014

[bjop12727-bib-0050] Tong, S. , Jia, N. , Luo, X. , & Fang, Z. (2021). The Janus face of artificial intelligence feedback: Deployment versus disclosure effects on employee performance. Strategic Management Journal, 42(9), 1600–1631. 10.1002/smj.3322

[bjop12727-bib-0051] Valdesolo, P. , & DeSteno, D. (2007). Moral hypocrisy: Social groups and the flexibility of virtue. Psychological Science, 18, 689–690. 10.1111/j.1467-9280.2007.01961.x 17680939

[bjop12727-bib-0052] Vogt, S. , Mohmmed Zaid, N. A. , El Fadil Ahmed, H. , Fehr, E. , & Efferson, C. (2016). Changing cultural attitudes towards female genital cutting. Nature, 538(7626), 506–509. 10.1038/nature20100 27732586

[bjop12727-bib-0053] von Schenk, A. , Klockmann, V. , & Köbis, N. (2023). Social preferences toward humans and machines: A systematic experiment on the role of machine payoffs. Perspectives on Psychological Science, 1–17. 10.1177/17456916231194949 PMC11720266 37751604

[bjop12727-bib-0054] Weiss, A. , Burgmer, P. , & Mussweiler, T. (2018). Two‐faced morality: Distrust promotes divergent moral standards for the self versus others. Personality and Social Psychology Bulletin, 44(12), 1712–1724. 10.1177/0146167218775693 29804510

[bjop12727-bib-0055] Yang, M. (2023). New York City schools ban AI chatbot that writes essays and answers prompts. *The Guardian* . https://www.theguardian.com/us‐news/2023/jan/06/new‐york‐city‐schools‐ban‐ai‐chatbot‐chatgpt

