# Supplementary material for: People have different expectations for their own versus others' use of AI‐mediated communication tools
Source: Br J Psychol. 2024 Sep 4;117(2):548–66. doi: 10.1111/bjop.12727 (PMC13051031; doi:10.1111/bjop.12727)
Supplement: Supplementary file 1 — Data S1. [file BJOP-117-548-s001.docx]

**S. Supplementary Information**

**S.1 Demographic moderation.** For participants in the ‘self’ condition^^[[1]](#footnote-0)^^, we explored whether expectations for use, judgements of acceptability, and opportunity seeking differ between ages, genders^^[[2]](#footnote-1)^^, and levels of education. Specifically, we assess whether usage expectations, acceptability, and the relationship between the two were similar across demographic factors of age, gender, and education?

To assess whether some groups intend to use AICTs more than others, we used a linear mixed model predicting ‘usage’ from age, gender, and education with participant and medium included as random intercept effects. The effect of gender was significant (*B* = 6.22, *95% CI* [0.48, 11.97], *t*(1102) =2.12, *p* = .034). Males expected to use AICTs (*eM* =41.88, *SE* = 6.28) more than females (*eM* = 35.66, *SE* = 6.28). The effects of age (*t*(1102) = 0.18, *p* =.861) and education were not significant (*F*(5,363) = 1.73, *p* = .125).

To assess whether some groups were more accepting of AICT use than others, we used a linear mixed model predicting ‘acceptability’ from age, gender, and education with participant and medium included as random intercept effects. The effect of gender was significant (*B* = 7.36, *95% CI* [1.95, 12.77], *t*(1102) =2.67, *p* = .008). Males were more accepting of AICTs (*eM* = 55.34, *SE* = 5.80) than females (*eM* = 47.98, *SE* = 5.80). The effects of age (*t*(1102) = 1.21, *p* = 0.225) and education were not significant (*F*(5,363) = 1.54, *p* = .173). Notably, the relationships between gender and usage, and gender and acceptability, are similar, suggesting that for both men and women their usage expectations may be aligned with what they deem acceptable (or not).

To assess whether the relationship between usage expectations and acceptability differed by demographics, we regressed ‘usage’ from ‘acceptability’ and the demographic variables (separately) with participant and medium included as random intercept effects (e.g., usage ~ acceptability*age). Reflecting our earlier analyses, acceptability had a significant positive effect on usage expectations in all models with demographics. However, in the model regressing usage on acceptability and education, there was an interaction effect of acceptability and education on usage. Those with tertiary education showed a stronger relationship between acceptability and usage expectations than did those without tertiary education. These findings suggest that compared to those with tertiary education, those without tertiary education show weaker increases in usage expectations as judgments of acceptability increase. This may be due to anticipating other barriers to uptake such as motivation, needs, or access. However, these analyses and interpretations are strictly exploratory and should be considered tentative until further research is conducted.

No other effects were significant, details for each model are provided here:

- Age: In the model with usage predicted by age and acceptability, the effect of acceptability was statistically significant and positive (B = 0.72, 95% CI [0.60, 0.85], t(1106) = 10.99, *p* < .001). The effect of age was statistically non-significant and negative (B = -0.11, 95% CI [-0.30, 0.07], t(1106) = -1.18, p = 0.237; Std. beta = -0.02, 95% CI [-0.07, 0.03]). The effect of accept × age was statistically non-significant and positive (B = 1.19e-03, 95% CI [-1.54e-03, 3.93e-03], t(1106) = 0.86, *p* = .391).
- Gender: In the model with usage predicted by gender (binary) and acceptability, the effect of acceptability was statistically significant and positive (B = 0.77, 95% CI [0.72, 0.83], t(1106) = 26.10, *p* < .001). The effect of gender [Male] was statistically non-significant and negative (B = -0.03, 95% CI [-5.80, 5.74], t(1106) = -0.01, p = .992). The effect of acceptability × gender was statistically non-significant and positive (B = 6.20e-03, 95% CI [-0.08, 0.09], t(1106) = 0.15, *p* = .883).
- Education: In the model with usage predicted by education (binary) and acceptability, the effect of acceptability was statistically significant and positive (B = 0.75, 95% CI [0.69, 0.80], t(1106) = 27.70, p < .001). The effect of education [Tertiary] was statistically non-significant and positive (B = 2.21, 95% CI [-3.57, 7.98], t(1106) = 0.75, p = .453). The effect of accept × education binary [Tertiary] is statistically non-significant and positive (B = 0.08, 95%CI [-4.11e-03, 0.16], t(1106) = 1.87, *p* = .062)

1. We included only those in the self condition because it was not clear how the participant’s own demographics might impact their expectations for others or how these findings should be interpreted. Future studies may benefit from examining these questions with a priori justifications. [↑](#footnote-ref-0)
2. Eleven participants identified as non-binary and three preferred not to answer the question about gender. Due to power and generalizability concerns, these participants were excluded from the analyses that included gender as a fixed effect. [↑](#footnote-ref-1)
